# Gaps in Current Cardiometabolic Risk Assessment: A Review Supporting the Development of the C.O.R.E. Indicator Model

**DOI:** 10.3390/jcm15020617

**Published:** 2026-01-12

**Authors:** Calogero Geraci, Giulio Geraci, Agostino Buonauro, Valentina Morello, Francesca La Rocca, Roberta Esposito

**Affiliations:** 1 Azienda Sanitaria Provinciale of Caltanissetta (ASP), Cardiology Unit, 93100 Caltanissetta, Italy; cgeragi81@gmail.com; 2 Cardiobesity Group, Italy; 3 Department of Internal Medicine, Umberto I Hospital, University of Enna “Kore”, 94100 Enna, Italy; giulio.geraci@unikore.it; 4 Santa Maria della Pietà Hospital, ASL Napoli 3 Sud, Nola, 80035 Naples, Italy; stino84@gmail.com; 5 Independent Nutrition Biologist, Private Practice, 93100 Caltanissetta, Italy; valentinamorello86@gmail.com; 6 Department of Clinical and Experimental Medicine, University of Catania, 95122 Catania, Italy; 7 Department of Clinical Medicine and Surgery, Federico II University Hospital, 80131 Naples, Italy

**Keywords:** obesity, cardiometabolic risk assessment, autonomic dysfunction, heart rate variability, metabolic flexibility, epicardial adipose tissue, visceral adiposity, functional capacity, multidomain risk stratification, preventive cardiology

## Abstract

Obesity is a multidimensional condition characterized by autonomic imbalance, metabolic inflexibility, impaired physical resilience, and ectopic adiposity, pathophysiological alterations that arise long before overt cardiometabolic disease becomes clinically detectable. Despite this, current cardiometabolic risk scores continue to rely predominantly on biochemical and anthropometric variables, such as BMI, waist circumference, glucose, and lipid levels. While these markers are practical, inexpensive, and validated across large population cohorts, growing evidence shows that they offer limited incremental predictive value and fail to capture early functional and structural abnormalities. The recent literature highlights the prognostic importance of autonomic dysfunction, reduced metabolic flexibility, diminished cardiorespiratory fitness, impaired muscular strength, and ectopic fat depots including visceral and epicardial adiposity, independently of the traditional anthropometric indices. The domains remain absent from traditional algorithms such as the Metabolic Syndrome criteria, the Framingham Risk Score, and SCORE2. As a result, cardiometabolic risk is frequently underestimated in key subgroups, including young adults with obesity, individuals with high visceral adiposity but normal BMI, those with subclinical myocardial dysfunction, and metabolically unhealthy normal-weight phenotypes. This narrative review synthesizes current evidence on obesity-related cardiometabolic impairment, highlights major gaps in established risk scores, and supports the conceptual development of the C.O.R.E. (Cardio-Obesity Risk Evaluation) Indicator Model—a hypothesis-generating, non-validated multidomain framework integrating autonomic, metabolic, functional, and structural markers to enable earlier risk phenotyping in future studies.

## 1. Introduction

Obesity represents one of the most relevant and rapidly growing public health challenges worldwide [1]. While excessive caloric intake remains a central driver of weight gain [2], modern sedentary behaviors, physical inactivity, and lifestyle changes significantly contribute to the global rise in obesity [3]. Socioeconomic and environmental conditions further modulate obesity risk through gene–environment interactions and possible epigenetic mechanisms [4].

Obesity, defined as an abnormal or excessive accumulation of adipose tissue that impairs health, is characterized not only by increased fat mass but also by profound adipose tissue dysfunction. In individuals with obesity, this dysfunction promotes chronic low-grade inflammation, dysregulated adipokine secretion, and neurohormonal activation—processes that are strongly implicated in cardiovascular disease (CVD), particularly in the development of cardiac damage, and in multiple obesity-related comorbidities [5]. Structural and functional cardiac alterations are common in obesity and vary across disease severity [6,7]. Despite the frequent preservation of left ventricular ejection fraction, advanced imaging techniques consistently reveal early subclinical myocardial dysfunction, including impaired myocardial strain [8], increased left ventricular mass, elevated diastolic filling pressures [7,9,10] and left atrial enlargement [11,12,13]. In parallel, obesity is associated with alterations in myocardial substrate utilization, characterized by a shift toward increased fatty acid oxidation and reduced glucose oxidation, which impairs cardiac efficiency and promotes heart failure development. In this context, epicardial adipose tissue (EAT) has emerged as a metabolically active organ capable of modulating cardiac structure, electrophysiology, and arrhythmogenic risk—particularly atrial fibrillation—through local inflammatory, oxidative, and fibrotic mechanisms. Importantly, these subclinical alterations frequently precede overt cardiovascular disease but are not routinely integrated into clinical risk stratification or decision-making algorithms [14]. Consequently, patients with obesity may be classified as low or intermediate risk despite the presence of early myocardial, autonomic, or structural impairment, resulting in delayed preventive or therapeutic interventions [15]. Moreover, EAT has emerged as a metabolically active organ capable of influencing cardiac structure and electrophysiology, particularly in the context of atrial fibrillation (AF), through inflammation, oxidative stress, fibrosis, and myocardial fat infiltration [16,17]. EAT is now recognized as a distinct visceral fat depot with local paracrine and vasocrine effects on the myocardium and coronary vasculature, with mechanistic links to inflammation, fibrosis, and cardiometabolic risk; consequently, it has been proposed as a clinically meaningful imaging biomarker and potential therapeutic target [17,18].

Additionally, the chronic inflammatory and pro-thrombotic milieu characteristic of obesity contributes to coronary atherosclerotic damage, to atrial myopathy and increased thromboembolic risk, which is often underestimated in clinical practice [7].

Taken together, these findings highlight the complex and multidimensional nature of cardiometabolic impairment in obesity. However, current cardiometabolic risk scores insufficiently capture early functional, autonomic, and anatomical alterations, as they rely predominantly on biochemical or anthropometric variables. This limitation translates into clinically relevant decision-making gaps, particularly in patients with subclinical myocardial dysfunction or autonomic impairment [19,20,21].

In this context, the C.O.R.E. framework is proposed as a physiology-based, multidomain phenotyping model aimed at identifying early cardiometabolic vulnerability, rather than as a disease-specific risk prediction tool for a single clinical endpoint.

This gap underscores the need for integrated, multidomain tools capable of assessing cardiometabolic risk from a functional, autonomic, and structural perspective, providing the conceptual foundation for the C.O.R.E. (Cardio-Obesity Risk Evaluation) framework proposed in this narrative review.

## 2. Limitations of Current Cardiometabolic Risk Scores

Despite significant progress in the prevention and management of metabolic and cardiovascular diseases, current cardiometabolic risk scores remain largely grounded in static biochemical and anthropometric measures. Variables such as BMI, waist circumference (WC), fasting glucose, and lipid profiles form the basis of most risk stratification systems, including the Metabolic Syndrome criteria, the Framingham Risk Score, SCORE2, and other widely adopted prediction algorithms [22]. These tools remain widely used in clinical practice because they are simple, inexpensive, easily reproducible, and deeply embedded in international guidelines and large-scale epidemiological studies. However, as recently discussed elsewhere, these traditional markers, although valuable for population-level risk estimation, offer limited incremental predictive value when combined with standard clinical variables and fail to capture early alterations that occur long before overt cardiometabolic disease becomes clinically apparent [23]. Specifically, current models do not incorporate key dimensions such as autonomic imbalance, metabolic inflexibility, impaired physical resilience, and structural adiposity, all of which are central to the pathophysiology of obesity-related cardiometabolic dysfunction.

As a result, traditional risk scores tend to categorize cardiometabolic risk in an overly simplistic manner and often underestimate risk in several clinical subgroups, including young adults with obesity, individuals with high visceral adiposity but normal BMI, patients with subclinical cardiac dysfunction, and metabolically unhealthy normal-weight individuals. These gaps underscore the limitations of static, phenotype-based tools and highlight the need for multidomain assessment frameworks capable of capturing the dynamic and multidimensional nature of cardiometabolic impairment.

### Traditional Risk Markers: Strengths and Limitations

While classical cardiometabolic markers continue to represent the backbone of risk assessment owing to several well-recognized strengths [24], a deeper mechanistic perspective is required to explain their limited sensitivity in early disease stages. These measures—including BMI, WC, blood pressure, fasting glucose, and lipid profiles—are inexpensive, easily obtained during routine clinical evaluations, and widely applicable across diverse healthcare settings [25]. Their predictive capacity is supported by extensive epidemiological research, with consistent associations between traditional markers and long-term cardiovascular morbidity and mortality [26]. Because of their simplicity, reproducibility, and strong epidemiological grounding, these parameters remain integral to public health strategies and primary prevention frameworks [27].

Despite these advantages, traditional cardiometabolic markers exhibit important limitations, particularly in the early detection of cardiometabolic dysfunction. Most existing risk scores—including the Metabolic Syndrome criteria, the Framingham Risk Score, and SCORE2—are based on static biochemical and anthropometric variables and therefore have limited ability to capture the dynamic physiological processes that characterize the initial stages of metabolic and cardiovascular disease [22,28]. In a recent analysis, Stefan and Schulze demonstrated that anthropometric markers (BMI, WC) and standard biochemical parameters (fasting glucose, lipid levels) provide limited incremental predictive value when added to clinical variables and do not identify early pathophysiological alterations such as autonomic dysfunction, reduced metabolic flexibility, or abnormalities in fat distribution—mechanisms central to obesity-related cardiometabolic deterioration [23].

Growing evidence highlights that autonomic dysfunction, characterized by reduced vagal tone and sympathetic predominance, represents one of the earliest indicators of cardiometabolic impairment and is strongly associated with insulin resistance, arrhythmogenesis, and increased cardiovascular mortality, yet remains entirely absent from current risk assessment tools [29,30]. Similarly, metabolic inflexibility, reflecting early mitochondrial and substrate-utilization abnormalities, cannot be detected through fasting glucose, HbA1c, or lipid profiles—biomarkers that primarily represent late-stage metabolic dysfunction [31]. Moreover, markers of physical resilience, such as cardiorespiratory fitness, submaximal exercise performance, and muscular strength, consistently predict cardiovascular and all-cause mortality but are not incorporated into conventional scoring algorithms [30].

Together, autonomic dysfunction, metabolic inflexibility, and impaired physical resilience represent interconnected early manifestations of cardiometabolic vulnerability that precede overt disease and remain largely invisible to traditional risk scores. These domains constitute the conceptual core of the C.O.R.E. (Cardio-Obesity Risk Evaluation) framework, providing the mechanistic rationale for a multidomain approach to cardiometabolic risk stratification.

However, beyond functional, autonomic, and metabolic domains, the pathophysiological mechanisms underlying early cardiometabolic deterioration in obesity extend well beyond what can be captured by static biochemical or anthropometric measures. A further critical limitation is the lack of assessment of structural adiposity. Visceral adipose tissue (VAT), epicardial adipose tissue (EAT), and ectopic fat infiltration within skeletal or cardiac muscle are strongly linked to inflammation, atrial myopathy, heart failure with preserved ejection fraction (HFpEF), and cardiometabolic disease progression. However, neither BMI nor WC adequately reflects these ectopic fat depots, resulting in frequent misclassification of cardiometabolic risk—particularly among individuals with obesity or metabolically unhealthy normal-weight phenotypes [32].

Taken together, these limitations explain why traditional cardiometabolic scores often underestimate risk in key clinical subgroups such as young adults with obesity, individuals with high visceral adiposity but normal BMI, patients with subclinical cardiac dysfunction, and metabolically unhealthy normal-weight individuals. This diagnostic gap reinforces the need for integrated, multidomain frameworks capable of identifying early alterations across autonomic, metabolic, functional, and structural domains of cardiometabolic health, with potential implications for earlier risk reclassification and preventive strategies.

## 3. Multidimensional Pathophysiology of Obesity-Related Cardiometabolic Impairment

Obesity is increasingly understood not as a simple consequence of excessive adiposity but as a complex, multisystem disorder involving early disturbances across autonomic, metabolic, functional, and structural domains. These alterations frequently precede overt clinical manifestations and act synergistically to drive cardiometabolic decline.

*Autonomic imbalance* represents one of the earliest and most consistent observed features of obesity-related cardiometabolic dysfunction. Reduced vagal tone, heightened sympathetic activation, and impaired heart rate variability have been documented even in metabolically “healthy” obese individuals and are strongly correlated with insulin resistance, systemic inflammation, arrhythmogenicity, and increased cardiovascular mortality [20,29]. These data highlight autonomic dysregulation as a subclinical yet clinically meaningful determinant of cardiometabolic vulnerability.

Simultaneously, *metabolic inflexibility*, defined as the impaired ability to switch between lipid and glucose oxidation, reflects early mitochondrial dysfunction and disrupted substrate utilization. An increase in adiposity is associated with reduced oxidative flexibility, accumulation of lipid intermediates, and impaired energetic efficiency, all of which contribute to insulin resistance and myocardial metabolic remodeling [31,33]. These abnormalities occur long before alterations in fasting glucose or lipid profiles become detectable, emphasizing the need for earlier metabolic markers.

Functional impairment further contributes to early cardiometabolic instability. Decreased cardiorespiratory fitness, suboptimal responses to submaximal exertion—such as alterations in DFA-α1 (the short-term scaling exponent derived from detrended fluctuation analysis of heart rate variability, reflecting autonomic–metabolic coupling during exercise)—delayed heart rate recovery, and reduced muscular strength are strong, independent predictors of cardiovascular and all-cause mortality [28,34]. These markers reflect a progressive deterioration in physiological resilience, an essential but largely unmeasured dimension of cardiometabolic health, and they often manifest before individuals with dysmetabolism develop clinically detectable obesity.

Finally, structural adiposity, particularly VAT and EAT, plays a direct mechanistic role in cardiometabolic deterioration [14,18]. These ectopic fat depots exhibit high inflammatory and endocrine/paracrine activity, contributing to atrial myopathy, HFpEF, ventricular remodeling, endothelial dysfunction, and systemic inflammation. Increased VAT and EAT burden—sometimes present even in individuals with normal BMI, further demonstrating that conventional anthropometric indices cannot accurately capture structural cardiometabolic risk [6,9,28,32]. Importantly, the early identification of these functional, autonomic, and metabolic alterations has relevant translational and preventive implications, as it may enable earlier risk reclassification and the implementation of targeted lifestyle, behavioral, or exercise-based interventions before irreversible cardiometabolic damage becomes clinically evident.

## 4. Existing Composite Cardiometabolic Scores and Current Gaps

Over the past decade, several composite indices have been introduced to improve cardiometabolic risk estimation beyond traditional anthropometric and biochemical parameters. Among the most prominent are the Visceral Adiposity Index (VAI), the Metabolic Score for Visceral Fat (METS-VF), and various Cardiometabolic Risk (CMR) Scores used in youth and general populations. (Table 1).

While these tools have shown improved risk discrimination and stronger associations with cardiometabolic outcomes compared with isolated anthropometric measures, their impact on individual risk reclassification and routine clinical decision-making has generally been modest, heterogeneous, and largely confined to research or population-based settings.

The VAI integrates WC, BMI, triglycerides, and high-density lipoprotein (HDL) cholesterol to estimate visceral adipose tissue dysfunction. Compared with simple anthropometric markers, VAI provides a better approximation of visceral fat activity; however, it remains limited to lipid and anthropometric parameters and does not capture the other pathophysiological alterations associated with adiposity [35,36]. As a result, VAI may refine risk estimation at the population level but rarely translates into actionable changes in individual clinical management.

The METS-VF score extends traditional metabolic indices by incorporating the METS-IR algorithm along with demographic variables to estimate visceral fat mass. While METS-VF improves the identification of insulin resistance and metabolic dysfunction, its focus remains restricted to the metabolic–visceral dimension, lacking integration of autonomic, functional, or ectopic fat markers [36,37]. Accordingly, its clinical utility remains mainly confined to metabolic risk profiling rather than comprehensive cardiometabolic risk reclassification.

Similar limitations apply to the CMR scores, predominantly used in youth research, which combine standardized z-scores of fasting glucose, lipid parameters, blood pressure, and adiposity indices. Although useful for evaluating clustered metabolic risk, these scores rely exclusively on static biochemical and anthropometric measures, providing limited insight into early physiological disturbances such as autonomic imbalance, metabolic inflexibility, or early ectopic adiposity [23,38]. Thus, their use is primarily descriptive and epidemiological, with limited implications for early preventive or therapeutic decision-making.

Across these tools, three major limitations consistently emerge. First, their biological scope remains narrow, as existing indices predominantly capture metabolic or adiposity-related abnormalities while overlooking autonomic, functional, and structural domains that are central to early cardiometabolic dysfunction. Second, the static nature of the variables included in these scores limits their ability to reflect dynamic physiological processes, such as heart rate variability, DFA-α1 patterns during exercise, muscular strength, or cardiorespiratory fitness. Finally, current scores lack an integrated resilience–burden model, as they quantify cardiometabolic risk without simultaneously incorporating positive markers of physiological resilience (e.g., heart rate variability, physical fitness, handgrip strength) together with negative markers of structural burden, such as epicardial and visceral adipose tissue distribution.

Given these limitations, there is a clear unmet need for a multidomain, physiologically grounded tool capable of capturing early autonomic dysregulation, metabolic inflexibility, functional impairment, and structural adiposity, dimensions that are fundamentally involved in the pathogenesis of obesity-related cardiometabolic disease. In response to this gap, we introduce the C.O.R.E. framework, a novel integrative and hypothesis-generating model to provide a more sensitive and comprehensive assessment of cardiometabolic vulnerability.

## 5. The C.O.R.E. Framework: Rationale, Domains, and Structure

The **C.O.R.E. (Cardio-Obesity Risk Evaluation)** framework was conceptualized to address these gaps by integrating complementary physiological and anatomical markers into a single, clinically applicable framework.

The C.O.R.E. Score should be regarded as a conceptual prototype and proof-of-concept framework, rather than a fully validated clinical risk prediction tool. Formal derivation, calibration, and outcome-based validation are beyond the scope of the present narrative review and represent key objectives for future research.

The C.O.R.E. model is based on two fundamental principles:

- Early cardiometabolic dysfunction develops across multiple interdependent domains—autonomic, metabolic, functional, and fat distribution.
- Risk stratification should reflect both protective capacities and pathological burdens—represented, respectively, by positive physiological indicators and negative structural markers of cardiometabolic deterioration.

Accordingly, the C.O.R.E. framework is composed of:

- Five domains (“resilience indicators”), which account for autonomic, functional, and metabolic variables, and to which a positive score above 0 was assigned.
- Two domains (“structural penalty indicators”), which account for the structural distribution of adipose tissue, and to which a negative score below 0 was conceptually assigned to reflect cumulative structural burden [14,36].

**Figure 1:**
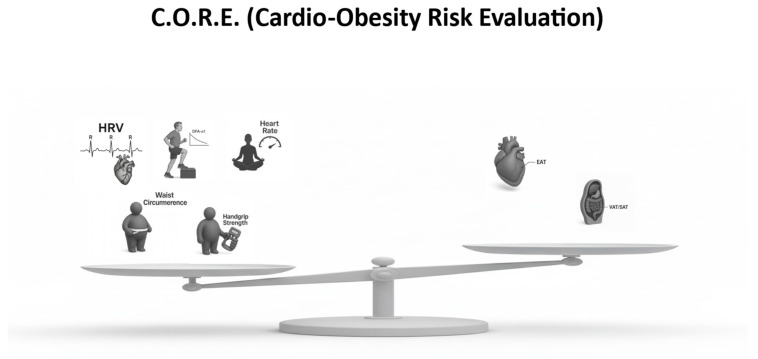
Conceptual architecture of the C.O.R.E. (Cardio-Obesity Risk Evaluation) framework, integrating positive resilience indicators (autonomic, metabolic, and functional domains) and negative structural penalty indicators (ectopic adiposity) into a multidomain cardiometabolic risk score.

This multidimensional structure is designed to enhance early detection of cardiometabolic vulnerability in individuals with obesity or at risk of obesity-related cardiometabolic disease. (Figure 1, Table 2 and Table 3).

The assignment of point ranges and the distinction between positive and negative scoring domains were guided by conceptual and methodological principles commonly adopted in multidomain clinical scoring systems. Similarly to established frameworks that integrate protective capacities and pathological burdens—such as frailty indices, cardiovascular risk scores, and resilience–burden models—the C.O.R.E. framework assigns positive scores to domains reflecting physiological resilience and adaptive capacity, and negative scores to domains representing cumulative structural and metabolic burden. The proposed point ranges are conceptually derived to reflect the relative physiological relevance and clinical interpretability of each domain and are intended to be hypothesis-generating, with future studies required for empirical calibration and validation.

### 5.1. Positive Domains (Resilience Indicators)

These domains reflect physiological systems whose integrity is indicative of cardiometabolic resilience. Higher scores across each domain reflect better cardiometabolic health.

**Resting Heart Rate Variability (HRV)**
Resting heart rate variability (HRV) reflects vagal modulation, autonomic balance, and systemic resilience and is therefore included as a positive resilience indicator [29].
**Submaximal Exercise Test with DFA-α1**
DFA-α1 assessed during submaximal exercise captures autonomic–metabolic coupling, metabolic flexibility, and aerobic efficiency, and is therefore included as a positive resilience indicator [39].
**Resting Heart Rate (RHR)**
Resting heart rate reflects autonomic tone and cardiorespiratory fitness, with lower values indicating greater physiological efficiency and resilience, and is therefore included as a positive resilience indicator [40].
**Waist Circumference (WC)**
Waist circumference reflects central adiposity and its associated metabolic burden and is therefore included as a positive resilience indicator when interpreted inversely, with lower values indicating greater cardiometabolic resilience [41,42].
**Handgrip Strength**
Handgrip strength reflects muscular fitness, metabolic efficiency, and global physiological vitality and is therefore included as a positive resilience indicator [43].

### 5.2. Negative Domains (Structural Penalty Indicators)

These indices reflect structural deterioration and adipose tissue dysfunction that directly increase cardiometabolic risk.

**Epicardial Adipose Tissue (EAT)**
Epicardial adipose tissue reflects ectopic fat accumulation and local inflammatory burden affecting cardiac structure and function and is therefore included as a negative structural penalty indicator [17].
**Abdominal Fat Distribution (VAT/SAT ratio and rectus fat infiltration)**
Abdominal fat distribution, including visceral-to-subcutaneous adipose tissue ratio and rectus muscle fat infiltration, reflects ectopic adiposity and structural metabolic burden and is therefore included as a negative structural penalty indicator [32].

### 5.3. C.O.R.E. Score Formula

To integrate these multidimensional components into a single composite risk metric, the following indices are defined:

- **Cardiometabolic Index (CmI)**

CmI = HRV + DFA−α1 + RHR + WC + Handgrip

*(Positive resilience indicators)*

- **Cardiometabolic Index- Ultrasound (CmI-U)**

CmI-U = EAT + VAT/SAT Index

where VAT/SAT Index reflects abdominal fat distribution.

*(Negative penalty indicators)*

- **Punteggio C.O.R.E.**

C.O.R.E. = CmI + CmI-U

The C.O.R.E. framework is intentionally conceived as a multidomain, physiology-driven model aimed at identifying early cardiometabolic vulnerability rather than predicting a single disease-specific outcome. Its primary application is early risk stratification in primary prevention settings, particularly among individuals with obesity or obesity-related metabolic dysfunction. Accordingly, future validation should focus on composite cardiometabolic outcomes—including incident ASCVD events, type 2 diabetes, HFpEF, atrial fibrillation, and progression of subclinical dysfunction—over short- to intermediate-term horizons, rather than conventional long-term (e.g., 10-year) risk prediction.

The proposed domains rely on standardized, guideline-consistent assessment methods commonly used in clinical and research practice. Specific acquisition protocols and cut-offs are intentionally not prespecified and are expected to be refined through prospective validation. While not intended as an immediately deployable clinical score, the modular structure of the C.O.R.E. framework is based on feasible and reproducible measures, allowing progressive adoption and validation across different healthcare contexts.

## 6. Operationalizing the C.O.R.E. Score: A Practical Clinical Guide

**This section operationalizes the C.O.R.E. framework by providing practical guidance on measurement, threshold interpretation, and scoring for each domain.** Each domain is presented in a standardized format to enhance feasibility and reproducibility across settings (Table 4).

### 6.1. Resting Heart Rate Variability (HRV)

Score Range: 0–30 points.

#### 6.1.1. Pathophysiological Rationale

Resting heart rate variability (HRV) is one of the most sensitive indicators of autonomic–metabolic integrity. RMSSD (Root Mean Square of Successive Differences), reflecting vagal modulation, and SDNN (Standard Deviation of NN Intervals), representing overall autonomic variability, capture the capacity of the autonomic nervous system to dynamically regulate cardiovascular function in response to internal and external demands. Reduced HRV signifies autonomic rigidity, heightened sympathetic activation, and impaired homeostatic adaptability, alterations strongly associated with insulin resistance, systemic inflammation, and cardiometabolic disease progression [19,29,44]. Importantly, reduced HRV can be observed even in individuals with normal anthropometric and biochemical profiles, making it a powerful early marker of visceral adiposity and latent metabolic dysfunction [32].

#### 6.1.2. Measurement Protocol

**Figure 2:**
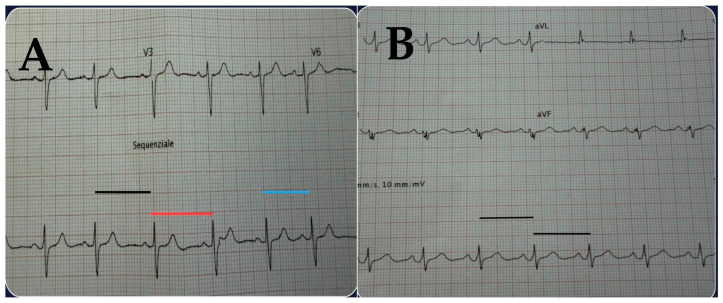
Resting Heart Rate Variability. From the standardized 5 min ECG assessment, two 10 s segments were selected to visually exemplify different autonomic profiles. Panel (**A**) illustrates a patient with preserved autonomic modulation, showing wider fluctuations in R–R intervals, represented by different colors: black, red, and blue. Panel (**B**) illustrates a patient with reduced autonomic variability. These tracings serve only as visual examples; all HRV values reported in the study derive from complete 5 min ECG recordings.

HRV should be recorded during a standardized 5 min resting measurement, with the patient supine in a quiet, thermoneutral room (22–24 °C) (Figure 2).

Patients should abstain from caffeine, nicotine, and alcohol for at least three hours before the test and avoid exercise for at least 12 h. The *gold* standard for measurement is ECG, although validated chest sensors (e.g., Polar H10 and Movesense) are acceptable alternatives for clinical practice. RMSSD is used as the primary metric, with SDNN evaluated as a complementary index.

#### 6.1.3. Interpretation and Thresholds

According to the normative ranges summarized in the literature [45], healthy adults typically exhibit RMSSD values around 30–40 ms in men and 32–42 ms in women, whereas values below 20 ms indicate a clinically meaningful impairment of vagal modulation. SDNN values above 50 ms reflect preserved global autonomic variability, while values below 30 ms are associated with increased cardiometabolic risk.

Within the C.O.R.E. framework, HRV scoring is intentionally conceived as a semi-quantitative, category-based approach rather than a continuous mathematical function. Points are assigned according to predefined physiological ranges that reflect autonomic integrity, early dysfunction, or overt impairment, rather than through proportional or exponential transformations.

Specifically, the proposed 0–30 point range is derived from RMSSD-centered categories as follows: values ≥ 30 ms are assigned the highest score range, reflecting preserved vagal modulation; values between 20 and 29 ms correspond to an intermediate score range, consistent with early autonomic strain; and values < 20 ms are assigned the lowest score range, indicating significant autonomic dysregulation. SDNN values are used as a complementary indicator to confirm global autonomic variability and reinforce classification in borderline cases.

This categorical scoring approach was deliberately chosen to enhance clinical interpretability and feasibility in routine practice. The development of continuous scoring functions, weighting schemes, or non-linear transformations will require prospective validation studies and outcome-based calibration, and therefore lies beyond the scope of the present review.

#### 6.1.4. Clinical Meaning

Low HRV is consistently associated with insulin resistance, metabolic syndrome progression, visceral adiposity accumulation [19,29,44], and cardiometabolic risk even in metabolically “normal” individuals [32]. Reduced vagal activity is also associated with cardiac arrhythmogenic vulnerability, heightened sympathetic tone, systemic inflammation, and increased all-cause mortality [44,46]. These associations underscore HRV as a non-invasive biomarker capable of identifying early cardiometabolic vulnerability far earlier than conventional markers such as fasting glucose, lipids, or blood pressure.

Although HRV measurements may vary across devices and signal-processing algorithms, the categorical scoring approach adopted within the C.O.R.E. framework is intended to reduce sensitivity to minor inter-device variability, provided that validated acquisition systems and standardized protocols are used.

#### 6.1.5. Interpretation and Thresholds

Within the C.O.R.E. framework, HRV contributes to the autonomic resilience domain as follows:

- **25–30 points: RMSSD ≥ 30 ms and SDNN ≥ 50 ms** → robust autonomic resilience

Preserved vagal modulation with intact global autonomic variability

- **15–24 points: RMSSD 20–29 ms and/or SDNN 30–49 ms** → early autonomic strain

Mild-to-moderate reduction in autonomic flexibility, compatible with early cardiometabolic vulnerability

- **0–14 points: RMSSD < 20 ms and/or SDNN < 30 ms** → marked autonomic dysfunction

Significant autonomic dysregulation associated with increased cardiometabolic risk

In cases of discordance between RMSSD and SDNN (e.g., preserved RMSSD with reduced SDNN, or vice versa), the lower category prevails, reflecting reduced overall autonomic robustness.

### 6.2. Submaximal DFA-α1 Step Test

Score Range: 0–30 points.

#### 6.2.1. Pathophysiological Rationale

The analysis of DFA-α1 (Detrended Fluctuation Analysis, short-term scaling exponent) of RR-interval dynamics during submaximal exercise provides a sensitive insight into the interaction between autonomic regulation, metabolic flexibility, and aerobic efficiency. Unlike resting HRV, DFA-α1 captures autonomic–metabolic coupling under controlled physiological stress, reflecting the capacity of the cardiovascular system to preserve complexity while adapting to increasing energetic demands.

In healthy individuals, heart rate dynamics maintain a fractal-like structure at low workloads (α1 ≈ 1.0), reflecting a balanced interplay between vagal withdrawal, sympathetic activation, ventilatory control, and efficient substrate switching. As workload increases, DFA-α1 declines in a predictable manner toward values around 0.75, marking the progressive loss of complexity associated with sympathetic predominance and the transition toward anaerobic metabolism. Gronwald et al. demonstrated that this decline closely tracks the aerobic threshold and represents an integrative marker of physiological resilience and autonomic–metabolic coordination [39].

#### 6.2.2. Measurement Protocol

The test is performed using a standardized 30 cm step or an equivalent low-intensity exercise protocol at a fixed cadence. RR-interval data are continuously recorded using validated chest-strap sensors (e.g., Polar H10 or Movesense). The primary measurement window corresponds to the steady-state phase at mild-to-moderate intensity (approximately 2–4 METs), a range in which autonomic complexity should remain preserved in metabolically healthy individuals. DFA-α1 is calculated over consecutive 2 min windows using validated algorithms and software.

#### 6.2.3. Interpretation and Thresholds

A DFA-α1 value ≥ 0.85 during low-to-moderate workloads indicates preserved physiological complexity and intact autonomic–metabolic coordination. Values between 0.75 and 0.84 suggest emerging dysregulation, reflecting early impairment in metabolic flexibility and autonomic adaptability. A premature decline of DFA-α1 below 0.75—particularly when occurring at workloads below 3–4 METs—signals an early loss of physiological resilience and disproportionate sympathetic dominance during minimal exertion.

Within the C.O.R.E. framework, the DFA-α1 domain is scored using a piecewise linear model (0–30 points) based on the steady-state DFA-α1 value recorded during submaximal exercise, with an additional penalty applied for premature loss of complexity at very low workloads:

- **25–30 points: α1 ≥ 0.85** → preserved physiological complexity and robust autonomic–metabolic resilience
- **15–24 points**: **α1 = 0.75–0.84** → early autonomic–metabolic dysregulation
- **0–14 points**: **α1 < 0.75 at low workloads (< 3–4 METs)** → impaired physiological resilience

This transparent scoring strategy preserves the biological meaning of DFA-α1 thresholds while allowing proportional risk stratification across a continuous physiological spectrum.

#### 6.2.4. Clinical Meaning

Individuals with obesity, metabolic syndrome, or insulin resistance frequently exhibit attenuated DFA-α1 responses, transitioning to low-complexity states at disproportionately low workloads [28,31]. This pattern reflects impaired mitochondrial efficiency, reduced oxidative capacity, and diminished vagal modulation during exercise, often preceding detectable abnormalities in fasting glucose, HbA1c, lipid profiles, or even resting HRV. DFA-α1 has also been linked to inflammatory burden, inefficient oxygen utilization, and exercise intolerance, supporting its role as an early and dynamic marker of cardiometabolic risk [33,39].

At present, DFA-α1 analysis may be more readily applicable in specialized clinical or research settings due to software and training requirements. Its inclusion within the C.O.R.E. framework reflects its strong physiological relevance, while recognizing that broader clinical implementation will require further dissemination, standardization, and training.

### 6.3. Resting Heart Rate (RHR)

Score Range: 0–30 points.

#### 6.3.1. Pathophysiological Rationale

RHR is a simple yet powerful biomarker of autonomic balance and cardiometabolic fitness. A lower RHR reflects robust parasympathetic tone, efficient myocardial energetics, and enhanced cardiorespiratory conditioning. Conversely, an elevated RHR is a marker of sympathetic overactivity, reduced vagal influence, and impaired metabolic health. Large population-based studies have consistently shown that RHR strongly predicts cardiovascular and all-cause mortality independently of conventional risk factors [40,47,48].

#### 6.3.2. Measurement Protocol

RHR is measured after at least 5 min of resting in a seated or supine position. The measurement should avoid recent caffeine intake, nicotine, or emotional stress. The average of 2–3 readings is recommended.

#### 6.3.3. Interpretation and Thresholds

Resting heart rate contributes up to 30 points to the C.O.R.E. score and is calculated using a *piecewise linear model* based on established prognostic thresholds. This approach was chosen to preserve clinical interpretability and avoid overfitting in the absence of prospective validation.

Scores are assigned as follows:

- **25–30 points: <70 bpm** → high cardiometabolic resilience
- **10–24 points: 70–79 bpm** → intermediate autonomic profile
- **0–9 points: ≥80 bpm** → increased autonomic and cardiometabolic risk

Within each category, points are allocated proportionally according to resting heart rate values, with lower heart rates receiving higher scores.

This linear scoring strategy reflects the continuous relationship between resting heart rate and cardiovascular risk reported in large epidemiological studies, while maintaining ease of application in routine clinical practice.

The proposed cut-offs were informed by guideline-consistent thresholds and population-based evidence describing a continuous increase in cardiovascular risk across resting heart rate categories. The piecewise linear approach was therefore selected to balance biological plausibility, clinical usability, and consistency with existing recommendations.

#### 6.3.4. Clinical Meaning

An elevated RHR correlates with insulin resistance, increased inflammatory load, impaired vascular compliance, and reduced cardiorespiratory fitness. It predicts incident diabetes, hypertension, and premature mortality independently of BMI or metabolic biomarkers.

### 6.4. Waist Circumference

Score Range: 0–10 points.

#### 6.4.1. Pathophysiological Rationale

Waist circumference (WC) remains one of the most robust, reproducible, and clinically practical markers of central adiposity and cardiometabolic risk. Unlike BMI, which does not differentiate between lean and fat mass and fails to capture fat distribution, WC directly reflects abdominal fat accumulation—a key determinant of insulin resistance, chronic low-grade inflammation, dyslipidemia, and cardiometabolic burden.

As emphasized in the IAS–ICCR Consensus Statement, WC should be considered a true clinical vital sign, given its strong and independent association with VAT, ectopic fat deposition, and cardiovascular morbidity and mortality [42]. Central adiposity exerts deleterious effects through inflammatory, endocrine, and lipotoxic mechanisms, directly contributing to metabolic dysfunction, myocardial remodeling, and vascular disease, even in individuals with normal BMI.

#### 6.4.2. Measurement Protocol

Waist circumference should be measured at the midpoint between the lowest rib and the iliac crest using a non-elastic measuring tape, with the patient standing upright, abdomen relaxed, and at the end of a normal expiration. Two consecutive measurements within 1 cm of each other should be obtained and averaged. Clinical interpretation should account for sex-specific and, when available, ethnicity-specific cut-offs, given known differences in fat distribution and cardiometabolic risk.

#### 6.4.3. Interpretation and Thresholds

According to the IAS–ICCR consensus, WC categories associated with increasing cardiometabolic risk are defined as follows:

- <80 cm in women/<94 cm in men → low central adiposity
- 80–88 cm in women/94–102 cm in men → intermediate risk
- ≥88 cm in women/≥102 cm in men → high central adiposity

These thresholds align closely with longitudinal trajectories of incident type 2 diabetes, hypertension, non-alcoholic fatty liver disease (NAFLD), HFpEF, and adverse cardiometabolic events.

Within the C.O.R.E. framework, WC contributes to the anthropometric domain through a piecewise linear scoring model (0–10 points) designed to reflect the progressive increase in cardiometabolic burden associated with central fat accumulation:

- **8–10 points: normal WC (<80 cm in women or <94 cm in men)** → minimal visceral adiposity burden
- **4–7 points: intermediate WC (80–88 cm in women or 94–102 cm in men)** → moderate central adiposity and early metabolic strain
- **0–3 points: elevated WC (≥88 cm in women or ≥102 cm in men)** → high visceral fat burden and increased cardiometabolic risk

This scoring strategy preserves the clinical interpretability of established WC cut-offs while allowing proportional integration of central adiposity into the multidomain C.O.R.E. score.

#### 6.4.4. Clinical Meaning

Waist circumference correlates strongly with visceral adiposity, hepatic steatosis, atherogenic dyslipidemia, impaired glucose tolerance, and systemic inflammatory activation [42]. Elevated WC is also associated with left atrial enlargement, diastolic dysfunction, coronary microvascular impairment, and increased risk of HFpEF, even in the absence of overt obesity.

Importantly, WC frequently identifies high-risk phenotypes such as metabolically unhealthy normal-weight (MUNW) individuals and thin-outside–fat-inside (TOFI) phenotypes, which are often misclassified as low risk by BMI-based or traditional cardiometabolic scores.

### 6.5. Handgrip Strength

Score Range: 0–20 points.

#### 6.5.1. Pathophysiological Rationale

Handgrip strength represents a robust, low-cost, and highly reproducible index of global muscular fitness, functional capacity, and cardiometabolic health. It correlates closely with skeletal muscle mass, neuromuscular integrity, mitochondrial efficiency, and insulin-mediated glucose uptake. Given that skeletal muscle is the largest insulin-sensitive tissue in the human body, reduced muscular strength reflects impaired metabolic reserve, anabolic resistance, and early disruption of whole-body energy homeostasis.

Beyond its musculoskeletal significance, handgrip strength integrates multiple cardiometabolic pathways, including chronic low-grade inflammation, autonomic imbalance, reduced oxidative capacity, and physical inactivity. Large population-based cohorts have consistently demonstrated that lower grip strength is independently associated with increased risk of type 2 diabetes, cardiovascular disease, frailty, hospitalization, and all-cause mortality, even after adjustment for BMI and traditional risk factors [43].

#### 6.5.2. Measurement Protocol

Handgrip strength is assessed using a calibrated handheld dynamometer (e.g., Jamar). The patient is seated or standing, with the shoulder neutrally positioned, elbow flexed at 90°, forearm in neutral position, and wrist slightly extended. Three maximal voluntary contractions are performed for each hand, separated by short rest intervals. The highest value obtained from the dominant hand is typically used for scoring.

To ensure clinical relevance and comparability, grip strength values should be interpreted using age- and sex-specific normative reference ranges derived from large population datasets.

#### 6.5.3. Interpretation and Thresholds

Population-based reference data identify three broad categories of functional status based on age- and sex-adjusted percentiles:

- Above the 50th percentile → preserved muscular and metabolic reserve
- Between the 25th and 50th percentile → early functional impairment
- Below the 25th percentile → high-risk profile consistent with sarcopenia, frailty, and adverse cardiometabolic prognosis

These thresholds show strong associations with incident type 2 diabetes, cardiovascular events, cancer mortality, disability, and reduced life expectancy [43].

Within the C.O.R.E. framework, handgrip strength contributes to the functional resilience domain through a percentile-based, piecewise linear scoring model (0–20 points) designed to reflect progressive loss of muscular and metabolic reserve.

- **16–20 points: above the age- and sex-specific 50th percentile** → preserved functional capacity and cardiometabolic resilience
- **8–15 points: between the 25th and 50th percentile** → intermediate strength and early functional decline
- **0–7 points: below the 25th percentile** → marker of functional impairment and a frailty-prone phenotype

This approach allows proportional integration of muscular fitness into the global C.O.R.E. score while maintaining strong epidemiological grounding and clinical interpretability.

#### 6.5.4. Clinical Meaning

Low handgrip strength reflects the convergence of several adverse biological processes, including mitochondrial dysfunction, impaired oxidative metabolism, systemic inflammation, autonomic dysregulation, and reduced habitual physical activity. Importantly, reduced strength often precedes overt weight gain or metabolic abnormalities and may be present in individuals with normal BMI or WC, thereby identifying early loss of physiological resilience that traditional anthropometric measures fail to detect.

As such, handgrip strength provides a simple yet powerful functional biomarker of cardiometabolic vulnerability and biological aging, with direct implications for risk stratification and intervention planning.

It should be acknowledged that handgrip strength may be influenced by conditions unrelated to cardiometabolic health, such as osteoarthritis, musculoskeletal limitations, or neurological disorders. Therefore, interpretation of this domain within the C.O.R.E. framework should take into account the individual clinical context, particularly in the presence of known non-metabolic causes of reduced muscular strength.

### 6.6. Epicardial Adipose Tissue (EAT)

Penalty Range: −20 to 0 points.

#### 6.6.1. Pathophysiological Rationale

Epicardial adipose tissue (EAT) is a metabolically active visceral fat depot located between the myocardium and the visceral pericardium. Unlike other adipose tissues, EAT is in direct anatomical and paracrine contact with the coronary arteries and atrial myocardium, lacking a fascial barrier. In conditions of excess accumulation, EAT undergoes a phenotypic shift toward a pro-inflammatory and profibrotic profile, characterized by the secretion of cytokines, adipokines, and oxidative mediators that directly promote atrial fibrosis, electrical remodeling, endothelial dysfunction, coronary atherosclerosis, and HFpEF pathophysiology [14,18].

For these reasons, EAT represents a mechanistically relevant marker of structural cardiometabolic burden, integrating adiposity-related inflammation with myocardial and vascular damage [49,50].

#### 6.6.2. Measurement Protocol

**Figure 3:**
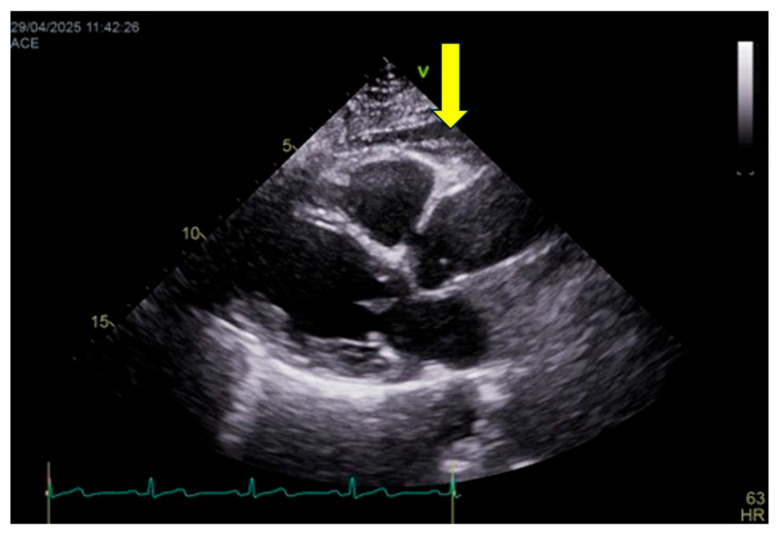
Echocardiographic Epicardial Adipose Tissue. The echocardiographic frame shows the hypoechoic adipose layer located between the visceral pericardium and the outer myocardial wall along the right ventricular free wall (arrow). EAT thickness is measured perpendicularly at end-systole, when the adipose layer is most clearly delineated.

EAT thickness is assessed primarily by transthoracic echocardiography (Figure 3), which represents the most practical and widely available imaging modality for routine clinical evaluation and longitudinal follow-up. EAT is measured as the echo-lucent space between the outer myocardial wall and the visceral pericardium, most commonly on the right ventricular free wall using parasternal long-axis and short-axis views. Measurements are obtained at end-systole—when EAT thickness is maximal—and averaged over at least three consecutive cardiac cycles.

Although computed tomography (CT) and magnetic resonance imaging (MRI) allow accurate volumetric quantification of epicardial fat and are considered reference techniques for research purposes, they are not incorporated into the C.O.R.E. score due to limited availability, radiation exposure (for CT), higher costs, and reduced feasibility for large-scale or repeated assessments. Accordingly, volumetric cut-offs are not included in the current scoring framework.

It should be acknowledged that echocardiography provides a linear estimate and does not fully capture total epicardial fat volume. Nevertheless, several studies have demonstrated a strong correlation between echocardiographic EAT thickness and volumetric measurements obtained by CT or MRI, as well as with cardiometabolic risk and cardiovascular outcomes. Thus, echocardiography is adopted in the C.O.R.E. model as a pragmatic compromise between diagnostic accuracy and clinical applicability, while recognizing its lower spatial resolution compared with tomographic techniques.

#### 6.6.3. Interpretation and Thresholds

Although no universally accepted cut-off exists, commonly used echocardiographic thresholds include:

- <5 mm: minimal or physiological EAT
- 5–7 mm: moderate EAT expansion
- >7 mm: severe EAT accumulation

These ranges have been consistently associated with increasing cardiometabolic risk, atrial arrhythmias, coronary artery disease, and HFpEF. While CT-based volumetric thresholds (e.g., >125 mL) have been linked to elevated risk, they are reported here for contextual reference only and are not used for scoring.

In the C.O.R.E. framework, EAT contributes as a negative (penalty) domain, reflecting structural cardiometabolic burden:

- **0 points penalty**: EAT < 5 mm → minimal structural burden
**−10 points**: EAT 5–7 mm → moderate epicardial adiposity
**−20 points**: EAT > 7 mm → severe epicardial adiposity

#### 6.6.4. Clinical Meaning

EAT is a particularly valuable marker in individuals who otherwise appear metabolically stable based on traditional parameters. Increased EAT may be present even when glucose, lipid profiles, and blood pressure are within normal ranges, frequently identifying TOFI (thin-outside–fat-inside) phenotypes and metabolically unhealthy normal-weight individuals. Elevated EAT burden correlates strongly with atrial fibrillation, coronary artery disease, ventricular remodeling, HFpEF, and systemic inflammatory activation, providing incremental risk information beyond BMI and waist circumference [7,16,17,41,50,51].

These EAT cut-offs are provided for conceptual and research purposes and should not be interpreted as definitive clinical thresholds. Their applicability may vary across populations and imaging protocols and will require prospective validation.

### 6.7. Abdominal Fat Distribution: VAT/SAT Ratio and Rectus Fat Ratio

Penalty Range: −20 to 0 points.

#### 6.7.1. Pathophysiological Rationale

Abdominal adipose distribution is one of the strongest determinants of cardiometabolic risk. A higher visceral-to-subcutaneous fat ratio (VAT/SAT) reflects deep, metabolically active adiposity associated with insulin resistance, hepatic steatosis, systemic inflammation, and ectopic lipid deposition [14,15]. The rectus abdominis fat ratio adds further information on local ectopic fat infiltration into the abdominal wall musculature, a marker of impaired muscle quality and metabolic dysfunction.

#### 6.7.2. Measurement Protocol

VAT and SAT can be quantified using ultrasound, CT, or MRI.

**Figure 4:**
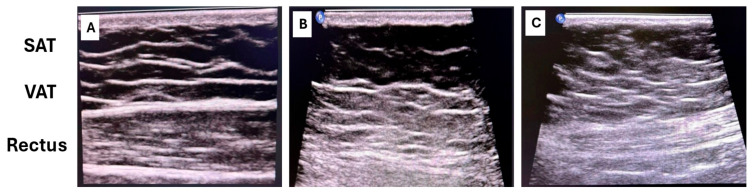
Ultrasound assessment of abdominal subcutaneous fat (SAT), visceral fat (VAT), and rectus muscle morphology across different cardiometabolic profiles. (**A**) Male, 45 years, no cardiometabolic disease. The ultrasound shows a well-organized layering pattern with clear distinction between SAT and VAT compartments, and a preserved, normally hypoechoic rectus abdominis muscle with appropriate thickness. (**B**) Male, 39 years, advanced cardiometabolic disease. The image demonstrates poor differentiation between SAT and VAT, with adipose tissue appearing predominantly hyperechoic and structurally disorganized. The rectus abdominis muscle shows marked hypotrophy and sclerosis, appearing diffusely hyperechoic on ultrasound. (**C**) Female, 70 years, early cardiometabolic impairment. A measurable distinction between SAT and VAT is still present, but the rectus abdominis muscle shows hypotrophy and increased echogenicity consistent with early sclerosis.

- Ultrasound represents a practical and low-cost approach suitable for routine clinical assessment [52]: VAT is measured as the distance between the posterior surface of the abdominal muscles and the anterior wall of the aorta or lumbar spine, while SAT is measured from the skin to the linea alba (Figure 4).
- CT and MRI provide precise volumetric quantification and remain the reference standards in research settings.

Rectus abdominis fat infiltration is evaluated using ultrasound by assessing muscle thickness and echotexture, or by CT cross-sectional imaging when available. Ultrasound-based assessment focuses on increased muscle echogenicity and reduced contractile tissue, which reflect fatty infiltration and fibrotic remodeling.

#### 6.7.3. Interpretation and Thresholds

VAT/SAT ratio.

Commonly used research benchmarks include:

- VAT/SAT < 0.4 → favorable fat distribution
- VAT/SAT 0.4–0.7 → intermediate cardiometabolic risk
- VAT/SAT > 0.7 → high visceral fat dominance

At present, no universally accepted quantitative cut-off values exist for rectus muscle fat infiltration. Therefore, assessment is based on qualitative or semi-quantitative criteria, including:

- increased muscle echogenicity compared with adjacent tissues,
- reduced muscle thickness,
- loss of normal fibrillar architecture.

Moderate-to-severe rectus fat infiltration—particularly when associated with increased echogenicity and muscle hypotrophy—has been consistently linked to insulin resistance, impaired glucose uptake, reduced metabolic flexibility, and adverse cardiometabolic profiles, independent of BMI and overall adiposity.

For this reason, rectus fat infiltration is considered a risk-amplifying structural marker, complementing the VAT/SAT ratio rather than replacing it.

Within the C.O.R.E. framework, abdominal fat distribution contributes as a structural penalty indicator as follows:

- **0 points penalty: low VAT/SAT + minimal rectus fat infiltration**
**–10 points: moderate visceral/ectopic fat distribution**
**–20 points: high visceral dominance + rectus fat infiltration**

#### 6.7.4. Clinical Meaning

Elevated visceral adiposity and rectus muscle fat infiltration are strongly associated with insulin resistance, NAFLD, systemic inflammation, and the progression of cardiometabolic disease, and have been increasingly linked to myocardial and atrial remodeling and HFpEF [7,14,42]. Importantly, these patterns are common in normal-BMI individuals with high cardiometabolic risk and therefore correct one of the major blind spots of traditional scores.

It should be acknowledged that the combined qualitative–quantitative assessment of VAT/SAT ratio and rectus muscle fat infiltration may introduce some degree of inter-observer variability, particularly when ultrasound-based methods are used. For this reason, the C.O.R.E. framework assumes standardized acquisition protocols and appropriate operator training to enhance reproducibility, while recognizing that further methodological harmonization and prospective validation are required.

### 6.8. Implementation Considerations and Sources of Measurement Variability

Although the C.O.R.E. framework emphasizes feasibility, several domains are subject to biological and technical variability that should be acknowledged. Autonomic measures derived from resting HRV are sensitive to respiratory patterns, ectopic beats, artifact handling, circadian influences, sleep-disordered breathing, arrhythmias, and commonly prescribed medications, particularly beta-blockers and other rate-modifying agents. Standardized acquisition protocols and careful clinical contextualization are therefore essential for reliable interpretation.

DFA-α1 assessment during submaximal exercise offers valuable insight into autonomic–metabolic coupling but may be influenced by baseline fitness heterogeneity, orthopedic limitations, device- and algorithm-specific differences, and incomplete standardization in obesity-focused clinical cohorts. Its application may therefore be most appropriate in settings with expertise in exercise physiology, with progressive dissemination as methodological harmonization advances.

Structural adiposity assessment also presents implementation challenges. Echocardiographic measurement of epicardial adipose tissue, while pragmatic, is operator-dependent and represents a linear surrogate of a volumetric construct. Reproducibility across centers is enhanced by consistent view selection, measurement timing within the cardiac cycle, standardized planes, and averaging strategies. Similarly, VAT/SAT assessment by ultrasound is clinically meaningful but technique-dependent, requiring attention to probe positioning, anatomical landmarks, respiratory phase, and operator training to minimize interobserver variability.

To accommodate these constraints, the C.O.R.E. framework is intentionally modular. When advanced imaging or specific functional testing is unavailable or contraindicated, alternative domains may still provide meaningful risk phenotyping. This tiered approach supports broader applicability while underscoring the need for future studies to formally assess interobserver reliability, quality thresholds, and standardized acquisition protocols.

## 7. Discussion

The evidence summarized in this narrative review reinforces the notion that obesity-related cardiometabolic disease is not merely a consequence of excess adiposity or abnormal biochemical markers, but rather a progressive, multisystem condition characterized by early and often silent disturbances across autonomic regulation, metabolic efficiency, physical resilience, and structural adiposity. Traditional cardiometabolic risk scores, such as the Metabolic Syndrome criteria, the Framingham Risk Score, and SCORE2, have undoubtedly played a central role in cardiovascular prevention, yet their reliance on static biochemical and anthropometric variables increasingly appears insufficient to capture the earliest phases of cardiometabolic vulnerability [22,23]. These tools were developed in a historical context where obesity was conceptualized primarily as excessive fat accumulation and metabolic disturbance. However, current evidence paints a far more complex pathophysiological scenario.

One of the most compelling insights emerging from recent literature is that cardiometabolic deterioration often begins several years before measurable changes in glucose, lipid levels, or blood pressure [23]. Early autonomic dysfunction and metabolic inflexibility are detectable even in metabolically ‘healthy’ obesity and are consistently linked to insulin resistance, systemic inflammation, visceral adiposity, and early myocardial metabolic remodeling [15,19,20,29,33]. These alterations reflect a loss of physiological flexibility that remains largely invisible to standard clinical metrics.

Functional impairments also precede clinical disease. Low cardiorespiratory fitness and reduced muscular strength are among the most powerful predictors of cardiovascular and all-cause mortality, outperforming many traditional risk factors [30,34]. Yet these dimensions of physiological resilience do not appear in any existing cardiometabolic scoring system. The consequence is a diagnostic gap in which individuals with preserved laboratory values but diminished physiological reserve may be incorrectly classified as low-risk.

Structural adiposity further complicates this picture. VAT and EAT are not inert depots but highly active endocrine and inflammatory organs [14,18]. Their expansion contributes to atrial remodeling, HFpEF, endothelial dysfunction, and disturbances in myocardial metabolism [6,9,28]. Importantly, increased VAT or EAT can occur even in people with normal BMI or with apparently favorable lipid profiles, classic examples of the “TOFI” phenotype (thin outside, fat inside) [14,17]. VAT/SAT imbalance, rectus muscle quality, and EAT thickness have all been associated with early cardiometabolic risk in multiple imaging studies [32,41,42,43,53]. Yet none of these structural markers are considered in widely used risk scores.

Collectively, this convergence of evidence underscores a fundamental limitation of current cardiometabolic assessment strategies: its focus on late, static manifestations of disease rather than early, dynamic physiological processes. Modern cardiometabolic pathology does not begin with overt hypertension, dyslipidemia, or glucose dysregulation; it begins with the loss of complexity, flexibility, and resilience across interconnected systems. Capturing these early abnormalities requires a multidomain framework that integrates autonomic markers, functional indicators, metabolic signals, and structural imaging findings.

The C.O.R.E. model emerges as a conceptual response to this unmet need. Rather than relying solely on risk factor accumulation, C.O.R.E. reframes cardiometabolic risk as a balance between resilience and structural burden. This dual-axis approach incorporates markers that reflect the body’s adaptive capacity, such as HRV, submaximal DFA-α1 dynamics, resting heart rate, waist phenotype, and muscular strength, alongside markers representing anatomical deterioration, including EAT and VAT/SAT distribution. Such integration allows for a more nuanced characterization of cardiometabolic status, particularly in individuals whose traditional risk scores fail to reveal subclinical dysfunction.

A physiology-based model may offer several advantages. First, it has the potential to identify at-risk individuals earlier, when interventions such as lifestyle modification, exercise therapy, or autonomic training can exert maximal benefit. Second, it provides clinicians with a more comprehensive picture of a patient’s cardiometabolic profile, enabling targeted therapeutic strategies rather than one-size-fits-all interventions. Third, many of the components of the C.O.R.E. Score, such as HRV, handgrip strength, and ultrasound-based VAT/SAT evaluation, are inexpensive, repeatable, and feasible in primary care or outpatient cardiology settings.

Despite its promise, the C.O.R.E. framework remains conceptual and requires rigorous validation. Prospective cohort studies are essential to determine whether its multidomain structure improves the prediction of cardiovascular events compared with existing risk scores. Moreover, the optimal weighting of each component will need refinement through statistical modeling and possibly machine learning approaches. Validation across diverse populations, different ages, sexes, ethnicities, and obesity phenotypes will also be critical, given the heterogeneity of obesity-related cardiometabolic disease.

Ultimately, the shift from static to dynamic, multidomain assessment mirrors a broader transformation in cardiovascular medicine. As our understanding of the early phases of cardiometabolic disease deepens, the tools we use to detect and stratify risk must evolve accordingly. By integrating autonomic, metabolic, functional, and structural domains into a unified framework, the C.O.R.E. Score represents a step toward more sensitive and physiologically grounded risk assessment. Whether it will achieve widespread clinical adoption will depend on future validation, but the rationale for moving beyond traditional metrics is increasingly compelling.

At present, the C.O.R.E. framework is hypothesis-generating and has not yet been tested in derivation or validation cohorts. Prospective studies across diverse populations are required to establish their predictive performance, calibration, and clinical utility.

## 8. Strengths and Limitations

The present review offers several strengths. First, it synthesizes evidence across traditionally separate domains, autonomic physiology, metabolic flexibility, functional capacity, and structural adiposity, providing an integrated perspective on the early pathophysiological mechanisms that precede overt cardiometabolic disease. This multidomain approach reflects the contemporary understanding that obesity-related cardiometabolic risk emerges from the interplay of neural, metabolic, and anatomical factors rather than from isolated biochemical abnormalities alone. Second, the review highlights limitations of existing risk scores using high-quality evidence from epidemiological, physiological, and imaging studies, strengthening the rationale for developing new assessment tools. Third, the C.O.R.E. model proposed here is grounded in clinically accessible biomarkers, HRV, submaximal DFA-α1 dynamics, resting heart rate, waist phenotype, handgrip strength, and basic ultrasound measures, enhancing its potential real-world applicability in both primary care and cardiometabolic settings.

However, several limitations must be acknowledged. This work is primarily conceptual and does not provide empirical validation of the C.O.R.E. Score. The relative weight of each domain has been defined based on pathophysiological plausibility and evidence of prognostic relevance, but future prospective studies are required to refine scoring thresholds and establish predictive accuracy. Additionally, while the review incorporates robust literature, heterogeneity across studies—differences in HRV methodology, exercise testing protocols, ultrasound techniques, and definitions of ectopic adiposity—may influence comparability and limit generalizability. The proposed model also assumes that all domains contribute additively to cardiometabolic risk, whereas interactions between autonomic, metabolic, and structural systems may be nonlinear and more complex. Finally, access to certain measures (e.g., EAT assessment or detailed VAT/SAT characterization) may vary across clinical settings, potentially limiting early implementation.

In addition, autonomic and functional domains included in the C.O.R.E. framework may be influenced by comorbid conditions (such as diabetes, cardiovascular disease, or neurological disorders) and by commonly prescribed medications (e.g., beta-blockers, antihypertensive agents, or antidepressants), which could act as potential confounders independent of cardiometabolic risk. These factors should be carefully accounted for in future validation studies and clinical implementations.

Despite these limitations, the review establishes a clear conceptual and scientific foundation for a multidomain cardiometabolic risk model. Future research should focus on validating the C.O.R.E. Score in diverse populations, examining longitudinal trajectories, and determining whether interventions targeting autonomic, metabolic, or structural domains can modify the score and improve clinical outcomes.

## 9. Future Perspectives

Initial validation should prioritize short-term and intermediate-term outcomes reflecting early cardiometabolic disease trajectories, before extending the framework toward longer-term clinical endpoints. Looking forward, several research directions could strengthen, validate, and expand the clinical utility of the C.O.R.E. model. These avenues can be conceptually organized into short-term validation priorities and subsequent medium-term expansion strategies aimed at enhancing scalability and physiological resolution.

As an immediate priority for near-term validation, future research should investigate the relationship between the C.O.R.E. score and markers of subclinical atherosclerosis, particularly carotid intima–media thickness (cIMT) and plaque burden. Carotid ultrasonography is an established surrogate marker of early vascular injury and a robust predictor of cardiovascular events [54,55,56]. Demonstrating that autonomic–functional markers such as HRV or DFA-α1, as well as structural markers such as EAT or the VAT/SAT ratio, correlate with carotid atherosclerotic changes would provide strong external validation for the multidomain logic underlying the C.O.R.E. score. Emerging evidence already suggests that impaired HRV is linked to early vascular aging and endothelial dysfunction [20,29], while visceral adiposity strongly correlates with carotid atherosclerosis and plaque presence [32]. Establishing these relationships within an integrated framework would clarify whether early autonomic and metabolic dysregulation precedes or parallels subclinical vascular disease development.

A second, closely related step concerns improving feasibility and scalability, particularly through the derivation of HRV metrics from standard 10 s ECG recordings. While 5 min recordings remain the gold standard for assessing RMSSD and SDNN, several studies suggest that short ECG segments can provide reliable approximations of vagally mediated HRV indices [57,58]. If short-ECG HRV metrics prove sufficiently concordant with 5 min recordings in obese and metabolically impaired populations, this would dramatically increase the scalability of autonomic assessment. Since 10 s ECGs are already ubiquitous in clinical care, this approach could allow seamless integration of the autonomic dimension of the C.O.R.E. score into routine cardiometabolic evaluation without requiring specialized equipment or prolonged assessments.

In a subsequent phase of model refinement, the biological depth of the C.O.R.E. framework could be expanded through the selective integration of circulating biomarkers, particularly adipokines. Adipose tissue–derived mediators such as adiponectin, leptin, resistin, and pro-inflammatory cytokines provide mechanistic insight into adipose tissue dysfunction, inflammatory burden, and metabolic regulation, thereby conceptually bridging structural adiposity (EAT, VAT) with downstream cardiometabolic impairment. Although adipokines are not included in the current version of the C.O.R.E. score due to limitations in assay standardization, biological variability, cost, and feasibility in routine clinical practice, their integration represents a promising future direction, particularly in research-oriented or specialized clinical settings. Future studies may evaluate whether selected adipokines add incremental prognostic value beyond imaging-based, autonomic, and functional domains. In parallel, the model could be expanded into an exercise-based framework, tentatively conceptualized as C.O.R.E.-X (Cardio-Obesity Risk Evaluation—Exercise). Exercise testing offers unique insights into physiological resilience and metabolic efficiency that cannot be captured at rest. Parameters such as estimated VO_2_max, HR recovery, ventilatory thresholds, and DFA-α1 behavior during graded exertion are among the strongest predictors of cardiovascular and all-cause mortality [34,39]. Integrating these functional signals with advanced metabolic biomarkers, including ApoB and LDL particle metrics, Lp(a), free fatty acids, fasting insulin/glucose ratios, and inflammatory indices such as the neutrophil-to-lymphocyte ratio (NLR), could provide a multidimensional phenotype that reflects not only cardiometabolic risk but also adaptive capacity, systemic metabolic strain, and cardiometabolic “reserve.” Recent evidence shows that markers like ApoB and Lp(a) carry substantial predictive value beyond traditional lipids [59], while metabolic biomarkers such as FFA (Free Fatty Acids) and glucose/insulin ratios reflect mitochondrial function and metabolic flexibility [31,33].

Together, these research directions outline a stepwise strategy for transforming the C.O.R.E. framework from a conceptual model into a validated and clinically actionable tool: first through external validation against subclinical vascular disease, then by enhancing feasibility for real-world implementation, and finally by expanding physiological depth through exercise-based and biomarker-enriched phenotyping.

To transition the C.O.R.E. framework from a conceptual model to a clinically informative tool, a structured validation strategy aligned with contemporary risk-model standards is required. Initial efforts should focus on derivation in well-characterized cohorts, followed by external validation in independent populations to assess generalizability across different clinical settings and obesity phenotypes.

Model performance should be evaluated across complementary dimensions, including discrimination (e.g., C-statistic), calibration (e.g., calibration plots and slopes), and clinical utility (e.g., decision curve analysis). In addition, the incremental value of the C.O.R.E. score beyond established cardiometabolic risk tools should be formally assessed using changes in discrimination metrics (ΔC-statistic) and, where appropriate, reclassification indices such as the net reclassification improvement (NRI) and integrated discrimination improvement (IDI).

All validation studies should pre-specify clinical endpoints and time horizons consistent with the intended application of the framework, with particular emphasis on short- to intermediate-term composite cardiometabolic outcomes. This structured roadmap provides a transparent and methodologically robust pathway for future validation, calibration, and potential clinical translation of the C.O.R.E. framework.

## 10. Conclusions

The evidence synthesized in this review highlights the need to rethink cardiometabolic risk assessment in the context of obesity. Traditional scores, grounded in static biochemical and anthropometric parameters, identify risk only after substantial metabolic and structural deterioration has occurred. In contrast, contemporary research demonstrates that early cardiometabolic impairment is characterized by autonomic imbalance, reduced metabolic flexibility, diminished functional capacity, and the accumulation of ectopic adipose depots, alterations that remain largely invisible to current clinical tools.

The C.O.R.E. framework proposed here addresses this diagnostic gap by integrating complementary domains of cardiometabolic health into a unified model. By combining autonomic markers (HRV, DFA-α1), functional indices (resting heart rate, handgrip strength), anthropometric profiling (waist circumference), and imaging-derived measures of ectopic adiposity (EAT, VAT/SAT distribution), C.O.R.E. reframes risk assessment as a balance between physiological resilience and structural burden.

This multidomain approach may enable earlier identification of vulnerable individuals, particularly those with discordant phenotypes such as metabolically unhealthy normal-weight subjects or young adults with subclinical dysfunction. Its components are accessible, non-invasive, and applicable in routine clinical settings, suggesting good potential for scalability.

However, C.O.R.E. remains a conceptual model and requires rigorous validation through prospective cohorts to define optimal weighting, thresholds, and predictive value relative to existing scores. Future studies should also examine whether targeted interventions can modify these domains and translate into improved clinical outcomes.

In summary, the C.O.R.E. model offers a physiologically grounded, multidimensional perspective on cardiometabolic risk, aligning with emerging evidence that early dysfunction is systemic rather than purely metabolic. Its validation could support more precise, anticipatory, and personalized approaches to cardiometabolic prevention in obesity.

If validated, this framework could inform earlier, physiology-based preventive strategies and support the evolution of cardiometabolic risk assessment guidelines toward a more anticipatory and personalized approach in obesity.

## Figures and Tables

**Table 1:** Comparison of Existing Cardiometabolic Scores vs. the C.O.R.E. framework.

Score/Model	Biological Domains Covered	Core Variables	Captures Autonomic Function?	Captures Functional Capacity/Fitness?	Captures Structural Adiposity (EAT, VAT)?	Major Limitations
**VAI**	Metabolic–Lipid	WC, BMI, TG, HDL	No	No	No	Restricted to lipid–anthropometric markers; does not assess autonomic or functional domains
**METS-VF**	Metabolic–Visceral	WC, glucose, TG/HDL, age	No	No	No	Focused solely on the metabolic–visceral axis; lacks functional and autonomic markers
**CMR Scores**	Metabolic–Anthropometric	Glucose, TG, HDL, BP, BMI, WC	No	No	No	Based on static variables; provides no insight into early autonomic or functional impairment
**C.O.R.E. Score**	**Autonomic–Metabolic–Functional–Structural**	**HRV, DFA-α1, RHR, WC, handgrip strength, EAT, VAT/SAT**	**Yes**	**Yes**	**Yes**	**Integrated multidomain** prototype **combining resilience indicators and structural burden; requires prospective derivation and validation.**

***Abbreviations:** BMI, body mass index; BP, blood pressure; C.M.R., cardiometabolic risk; C.O.R.E., cardio-obesity risk evaluation; DFA-α1, detrended fluctuation analysis alpha-1; EAT, epicardial adipose tissue; HDL, high-density lipoprotein; HRV, heart rate variability; METS-VF, Metabolic Score for Visceral Fat; RHR, resting heart rate; SAT, subcutaneous adipose tissue; TG, triglycerides; VAI, visceral adiposity index; VAT, visceral adipose tissue; WC, waist circumference.*

**Table 2:** Official C.O.R.E. Table—Domains, Range, Meaning.

Domain	Category	Range	Direction	Physiological Meaning
**HRV (RMSSD/SDNN)**	Positive	0–30	Higher = better	Autonomic resilience
**DFA-α1 Step Test**	Positive	0–30	Higher = better	Metabolic and aerobic efficiency
**RHR**	Positive	0–30	Lower = better	Autonomic/metabolic fitness
**WC**	Positive	0–10	Lower = better	Central adiposity burden
**Handgrip Strength**	Positive	0–20	Higher = better	Functional/physiological reserve
**EAT**	Negative	−20–0	Higher = worse	Structural cardiac adiposity
**VAT/SAT + Rectus Fat**	Negative	−20–0	Higher = worse	Abdominal/ectopic adiposity

***Abbreviations** DFA-α1, detrended fluctuation analysis alpha-1; EAT, Epicardial Adipose Tissue; HRV, Heart Rate Variability; RMSSD, Root Mean Square of Successive Differences; RHR, Resting Heart Rate; SAT, Subcutaneous Adipose Tissue; SDNN, Standard Deviation of NN Intervals; VAT, Visceral Adipose Tissue; VAT/SAT, Visceral Adipose Tissue/Subcutaneous Adipose Tissue; WC, Waist Circumference.*

**Table 3:** Proposed Interpretation and Preliminary Thresholds (Pending Validation).

C.O.R.E. Score Range	Risk Interpretation	Predominant Phenotype
≥80	Normal/Resilient	High autonomic–functional–metabolic integrity
40–79	Intermediate cardiometabolic risk	Early dysfunction/mixed phenotype
<40	High cardiometabolic risk	Structural–autonomic–metabolic deterioration

*Thresholds are hypothesis-generating; clinical cut-offs require prospective validation.*

**Table 4:** Operationalization of the C.O.R.E. Score: Clinical Measurement, Key Cut-Offs, and Interpretation.

Domain	Clinical Measurement	Key Cut-Offs (C.O.R.E.)	Clinical Interpretation
Resting HRV	5 min supine ECG recording; RMSSD and SDNN analysis; avoid caffeine/nicotine/exercise before testing.	RMSSD ≥ 30 ms (high resilience); 20–29 ms (early autonomic dysregulation); <20 ms (high autonomic risk)	Early autonomic marker linked to vagal tone, inflammation, and cardiometabolic vulnerability
DFA-α1 Step Test	Submaximal step or treadmill test; steady-state DFA-α1 analysis during exercise.	α1 ≥ 0.85 (preserved complexity); 0.75–0.84 (mild dysfunction); <0.75 (impaired metabolic–autonomic coupling)	Identifies metabolic inflexibility, low CRF, and early mitochondrial inefficiency
RHR	Seated or supine HR after 5–10 min rest; validated HR monitor.	<70 bpm (optimal); 70–79 bpm (moderate risk); ≥80 bpm (high sympathetic burden)	Predictor of mortality risk, autonomic imbalance, and reduced fitness
WC	Measurement at midpoint between iliac crest and lower ribs; end-expiration.	Sex-specific IAS/ICCR thresholds	Surrogate marker of visceral adiposity; identifies central obesity not captured by BMI
Handgrip Strength	Handheld dynamometer; dominant hand; best of three trials.	Age- and sex-adjusted quintiles; lowest quintile = highest risk	Marker of muscular reserve, metabolic health, frailty, and mortality
EAT	Echocardiography (linear thickness); CT/MRI (volumetric assessment)	<5 mm: physiological EAT 5–7 mm: moderate EAT >7 mm: severe EAT	Local indicator of inflammation; associated with AF, HFpEF, and TOFI phenotype
VAT/SAT + Rectus Fat Ratio	Ultrasound or CT/MRI	VAT/SAT <0.4/0.4–0.7/>0.7; rectus fat infiltration graded qualitatively (none/mild vs. moderate–severe) to refine penalty category	Detects ectopic adiposity, insulin resistance, and early metabolic dysfunction

*Provides a concise clinical summary of each C.O.R.E. domain, including recommended measurement techniques, scoring thresholds, physiological significance, and supporting evidence. **Abbreviations:** AF, Atrial Fibrillation; CRF, Cardiorespiratory Fitness; CT, Computed Tomography; DFA, Detrended Fluctuation Analysis; DFA-α1, Detrended Fluctuation Analysis alpha-1; EAT, Epicardial Adipose Tissue; HFpEF, Heart Failure with Preserved Ejection Fraction; HR, Heart Rate; HRV, Heart Rate Variability; IAS/ICCR, International Atherosclerosis Society/International Chair on Cardiometabolic Risk; MRI, Magnetic Resonance Imaging; RMSSD, Root Mean Square of Successive Differences; RHR, Resting Heart Rate; SAT, Subcutaneous Adipose Tissue; SDNN, Standard Deviation of NN Intervals; TOFI, Thin Outside Fat Inside; VAT, Visceral Adipose Tissue; VAT/SAT, Visceral Adipose Tissue/Subcutaneous Adipose Tissue; WC, Waist Circumference. All cut-offs reported in Table 4 are hypothesis-generating and intended for conceptual and research purposes. Their applicability may vary according to population characteristics, measurement protocols, and clinical context, and therefore requires population-specific calibration and prospective validation.*

## Data Availability

The original contributions presented in the study are included in the article, further inquiries can be directed to the corresponding authors.
